# Cerebral toxoplasmosis in the twenty-first century

**DOI:** 10.1097/QAD.0000000000004356

**Published:** 2025-09-24

**Authors:** Charlotte M. van Deuzen, Bart J.A. Rijnders, Hannelore I. Bax, Casper Rokx, Theodora E.M.S. de Vries-Sluijs, Carolina A.M. Schurink, Jan L. Nouwen, Mariana de Mendonça-Melo, Adam A. Anas, Eric C.M. van Gorp, Lennert Slobbe, Jaap J. van Hellemond, Els van Nood

**Affiliations:** a Department of Medical Microbiology & Infectious Diseases; b Department of Internal Medicine, Erasmus University Medical Centre, Rotterdam, the Netherlands.

**Keywords:** AIDS, AIDS-related opportunistic infection, cerebral toxoplasmosis, HIV, treatment outcome

## Abstract

**Background:**

Cerebral toxoplasmosis is a common opportunistic infection in people with HIV (PWH), associated with high morbidity and mortality. It is unclear how clinical characteristics, treatment response and long-term clinical outcomes in PWH with cerebral toxoplasmosis have changed due to improved treatment of HIV.

**Methods:**

This single-centre retrospective observational cohort study of PWH with cerebral toxoplasmosis included patients over almost 25 years.

**Results:**

Sixty-three eligible patients were identified. Most patients were late presenters presenting with headache and neurological symptoms. Overall survival was 79% over a mean follow up of 15 years. Seventy-three percent of deaths occurred within the first year after diagnosis. Almost 10% of patients experienced residual impairments.

**Conclusion:**

An earlier diagnosis of HIV reduces the incidence of cerebral toxoplasmosis due to timely initiation of combination antiretroviral therapy (cART) and anti-*Toxoplasma* prophylaxis. High index of suspicion by clinicians is vital to timely start anti-*Toxoplasma* therapy. If treated correctly and timely, overall survival is high.

## Introduction

Cerebral toxoplasmosis is one of the most common causes of central nervous system infection in people with HIV (PWH) who progress to AIDS and is associated with high morbidity and mortality [1–3]. Toxoplasmosis is one of the most common infections in humans. It is estimated that about one-third of the global population is infected with *Toxoplasma gondii* during life [4,5]. However, the prevalence of infection varies substantially amongst different geographic regions and different ethnic groups within one region. Higher seroprevalence exists in lower income countries with seroprevalences up to 80% in certain parts of European, Latin American, and African countries [5–7]. *T. gondii* seroprevalence among people with AIDS mirrors the rate of the general population [3,7].

Humans can be infected by *T. gondii* through ingestion of infectious oocysts, most commonly from the environment contaminated with feline faeces or by eating undercooked meat from a *T. gondii*-infected animal [5,8]. In immunocompetent patients, the parasite disseminates throughout the body after oral ingestion and commonly causes an asymptomatic infection, after which it remains dormant for the life of the host in muscles and brain tissue. However, latent *T. gondii* infection can reactivate if the host becomes immunocompromised, such as in people with AIDS [9]. The lifetime probability of a latent *T. gondii* infection reactivation is up to 30% in people diagnosed with AIDS, of whom the HIV infection remains untreated and who do not receive effective anti-*Toxoplasma* prophylaxis [10]. In these patients, the most common clinical presentation of reactivation is cerebral toxoplasmosis. Clinical presentation often consists of focal encephalitis with headache, fever, and (severe) neurological or behavioural changes [1,3,8,11,12]. Most people respond well to anti-*Toxoplasma* therapy. However, neurological long-term complications can have a significant impact on subsequent quality of life [11–13].

Cerebral toxoplasmosis has been described as an AIDS-defining illness since the 1980s [14]. Multiple trials from that era describe the clinical characteristics and response to treatment at that time. There have been multiple advancements in the treatment of HIV since then. In the mid-1990s, two-drug combinations showed superiority to a single-drug regimen, and triple-drug therapy was introduced later that decade. These improvements in HIV treatment have greatly improved survival and decreased the incidence of AIDS-defining illnesses such as cerebral toxoplasmosis. With the decline in prevalence, recent larger studies on cerebral toxoplasmosis focus primarily on prophylaxis and treatment modalities, both in PWH and other immunocompromised patients, rather than clinical presentation [15,16].

In this observational study, we describe the clinical characteristics, response to treatment and long-term clinical outcomes of PWH that presented with cerebral toxoplasmosis at a large university medical centre in the Netherlands in the twenty-first century.

## Methods

This is a single-centre retrospective observational cohort study of PWH with cerebral toxoplasmosis at a 1125-bed university medical centre in the Netherlands, that retrospectively included patients in the modern combination antiretroviral therapy (cART) era from 1 January 2000 until 1 July 2024.

### Study design

Eligible patients were identified from two separate databases. The university medical centre's medical microbiology laboratory database was searched for *T. gondii*-positive PCR samples from PWH. Only used data from PWH in care in our centre who were already included in the Dutch national HIV database, Stichting HIV Monitoring (SHM), were used.

In the Netherlands, HIV care is provided by 26 designated treatment centres. As an integral part of HIV care, SHM is responsible for prospectively collecting data on HIV and comorbid conditions, including cerebral toxoplasmosis, from PWH living in the Netherlands and receiving care in one of these treatment centres. At its inception, this cohort was approved by the institutional review boards of all participating centres. Patients provide informed consent on collection of their data. Data are pseudonymized and made available to investigators for scientific purposes. All data were collected and managed in compliance with the Dutch General Data Protection Regulation (GDPR).

Patients below the age of 18 years or patients with noncerebral toxoplasmosis were excluded. For all included patients, medical files and computerized records were reviewed to determine demographic characteristics, clinical characteristics, treatment, and clinical outcomes.

For the suspicion of the diagnosis of cerebral toxoplasmosis a compatible clinical syndrome with suspect radiologic and positive microbiology findings, and clinical response after 14 days of anti-*Toxoplasma* therapy was needed. Cerebral toxoplasmosis was considered proven with presence of *T. gondii*-specific IgG antibodies in serum combined with presence of *T. gondii* in brain tissue or liquor (histology or PCR), or a positive Goldman–Wittmer coefficient (GWC). In our centre, the GWC is used to indicate specific enrichment of *Toxoplasma*-specific antibodies in the cerebrospinal fluid (CSF). In combination with the reported albumin index that provides information on the blood–brain barrier function, the GWC can be used to diagnose central nervous system (CNS) toxoplasmosis. Thereby, it is an alternative for the Reiber coefficient. The GWC and the Reiber coefficient both calculate the ratio of pathogen-specific antibodies in blood and liquor, but the GWC is more often used to diagnose ocular infections, such as uveitis, while the Reiber coefficient is used to diagnose CNS localization. A GWC greater than 3 is considered as a serological indication for CNS toxoplasmosis, where the threshold for the Reiber coefficient holds greater than 1.5. Cerebral toxoplasmosis was considered probable if liquor or brain tissue could not be obtained.

### Statistical analysis

Descriptive statistics were used, and continuous variables were summarized as means, medians, and interquartile ranges. Categorical variables were summarized as counts and percentages. Missing values were omitted from all summaries or explicitly stated.

## Results

### Patient characteristics

**Fig. 1:**
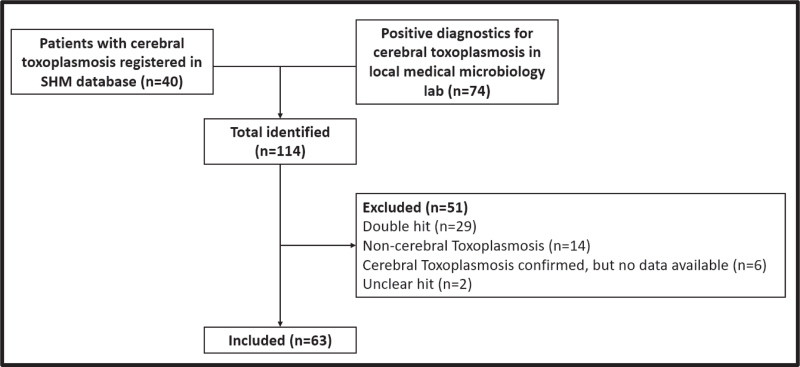
Flowchart of identification and inclusion of patients from the national Dutch HIV database from Stichting HIV Monitoring and the local medical microbiology laboratory database.

In total, 114 eligible patients were identified, of whom 63 (64%) patients could be included as these patients fulfilled the definition of either proven or probable cerebral toxoplasmosis (Fig. 1).

Of the 63 included patients, most were men (*n* = 35, 56%). One-third was born in the Netherlands (Table 1). Mean age at the time of cerebral toxoplasmosis diagnosis was 39 years. In approximately half of patients (*n* = 34, 54%), cerebral toxoplasmosis was diagnosed as the first presenting sign of HIV. These patients had a mean CD4^+^ T-cell count of 0.05 (0.02–0.06) × 10^9^/l. In 18 (28%) patients, cerebral toxoplasmosis developed months to years following their HIV diagnosis. These patients were either not on cART yet or had interrupted therapy. In eight (13%) patients, cerebral toxoplasmosis presented as unmasking immune reconstitution inflammatory syndrome (IRIS) after start of cART after a mean time of 2 months. Within 4–6 months of treatment, the mean CD4^+^ T-cell count in these patients increased from 0.05 to 0.26 × 10^9^/l, compared to the mean increase of CD4^+^ T-cell count from 0.05 to 0.18 × 10^9^/l in patients without IRIS.

**Table 1:** Demographic characteristics of people with HIV with cerebral toxoplasmosis.

Demographic characteristics (*n* = 63)	
Male [*n* (%)]	35 (56)
Born in the Netherlands [*n* (%)]	18 (29)
Dutch residency [*n* (%)]	55 (87)
Ethnicity	
Black [*n* (%)]	27 (43)
Caucasian [*n* (%)]	24 (38)
Latino [*n* (%)]	8 (13)
Other [*n* (%)]	4 (6)
Age at cerebral toxoplasmosis diagnosis, mean [IQR] (years)	39.0 [31.0–46.0]
HIV RNA at cerebral toxoplasmosis diagnosis, mean [IQR] (E+05 geq/ml)	1.97 [1.00–1.47]
CD4^+^ T-cell count at cerebral toxoplasmosis diagnosis, mean [IQR] (10^9^/l)	0.05 [0.02–0.06]
Time relationship between cerebral toxoplasmosis diagnosis and HIV diagnosis	
Cerebral toxoplasmosis first presenting sign of HIV [*n* (%)]	34 (54)
Cerebral toxoplasmosis whilst known HIV-positive [*n* (%)]	18 (28)
Cerebral toxoplasmosis as IRIS [*n* (%)]	8 (13)
Time between start cART and possible IRIS, mean [IQR] (months)	10.1 [0.5–3.5]
Relation cerebral toxoplasmosis and HIV unknown [*n* (%)]	3 (5)
Age at HIV diagnosis, mean [IQR] (years)	39.0 [28.3–46.0]
HIV type 1 [*n* (%)]	61 (97)
HIV RNA at HIV diagnosis, mean [IQR] (E+05 geq/ml)	1.86 [1.00–1.47]
CD4+ T-cell count at HIV diagnosis, mean [IQR] (10^9^/l)	0.08 [0.02–0.08]

IQR, interquartile range; IRIS, immune reconstitution inflammatory syndrome.

In three (5%) patients, timing of cerebral toxoplasmosis diagnosis was uncertain in relation to timing of their HIV diagnosis.

Clinical characteristics are depicted in Table 2. Most reported symptoms were headache (*n* = 35, 56%), paresis (*n* = 18, 29%), fever (*n* = 18, 29%), Additionally, more than one-third of patients, complained of other nonspecific nonneurologic symptoms, such as fatigue, malaise, nausea, vomitus, and weight loss.

**Table 2:** Clinical characteristics at initial presentation of cerebral toxoplasmosis diagnosis in people with HIV.

Clinical characteristics (*n* = 63)	
Headache [*n* (%)]	35 (56)
Paresis [*n* (%)]	18 (29)
Fever [*n* (%)]	18 (29)
Walking impairment [*n* (%)]	17 (27)
Speech impairment [*n* (%)]	14 (22)
Altered consciousness [*n* (%)]	12 (19)
Epilepsy [*n* (%)]	11 (18)
Other neurologic symptoms^a^ [*n* (%)]	33 (52)

a Other neurologic consisted of, but were not limited to, amnesia, changed behaviour, confusion, tremble, vertigo, and visual impairment.

### Diagnosis

Imaging was performed in all patients. Computed tomography (CT) scan or MRI scan was performed in 76% (*n* = 48) and 52% (*n* = 33) of patients, respectively, and for 24 (34%) patients, both imaging techniques were performed.

The number of lesions between individuals varied greatly. CT scan showed at least 10 lesions in 16 (33%) patients, between 2 and 9 lesions in 15 (31%) patients, and only a single lesion in 14 (29%) patients. On the CT scans of only two (5%) patients, no lesions were seen. On the CT scan of one (2%) patient, the number of lesions was not described, and images were not available. The distribution pattern of lesions among the patients examined by MRI scan was similar to that of CT scanning with contrast. However, in patients who got a CT scan without contrast and an MRI scan (*n* = 8, 13%), the number of lesions differed greatly in three patients, with more than 10 lesions on MRI scan with only a single lesion on CT scan without contrast.

Peri-lesional oedema was observed in most patients: in 86% (*n* = 38) by CT scan and in 90% (*n* = 28) by MRI scan. Follow-up imaging was performed in 88% (*n* = 55) of patients and showed improvement in more than 50% of patients.

*Toxoplasma* antibody determination (serology) in blood was available in most patients (*n* = 54, 86%) (Table 3). All *Toxoplasma* IgM antibody (index) tests were negative, whereas all but one *Toxoplasma* IgG antibody tests were positive with a mean concentration of 773 (197–1118) IU/ml. *Toxoplasma* PCR on blood was not commonly performed.

**Table 3:** Diagnostic characteristics of cerebral toxoplasmosis diagnosis in people with HIV.

Diagnostic characteristics (*n* = 63)	
Imaging performed [*n* (%)]	63 (100)
CT scan performed [*n* (%)]	48 (76)
Contrast used [*n* (%)]	32 (68)
Number of lesions seen	
0 [*n* (%)]	2 (5)
1 [*n* (%)]	14 (29)
2–9 [*n* (%)]	15 (31)
≥10 [*n* (%)]	16 (33)
Peri-lesional oedema [*n* (%)]	38 (86)
Follow-up CT scan performed [*n* (%)]	42 (88)
Time until follow-up CT scan performed, mean [IQR] (days)	19 [11–20]
Reduction of lesions in follow-up CT scan [*n* (%)]	24 (50)
MRI scan performed[*n* (%)]	33 (52)
Number of lesions seen	
0 [*n* (%)]	2 (6)
1 [*n* (%)]	6 (18)
2–9 [*n* (%)]	9 (28)
≥10 [*n* (%)]	16 (48)
Peri-lesional oedema [*n* (%)]	28 (90)
Follow-up MRI scan performed [*n* (%)]	15 (46)
Time until follow-up MRI scan performed, mean [IQR] (days)	24 [10–28]
Reduction of lesions in follow-up MRI scan [*n* (%)]	8 (53)
CT scan and MRI- scan both performed [*n* (%)]	24 (34)
*Toxoplasma* antibody determination (serology) in blood performed [*n* (%)]	54 (86)
*Toxoplasma* IgM antibody determination (index) performed [*n* (%)]	53 (98)
Positive [*n* (%)]	0 (0)
Negative [*n* (%)]	53 (100)
*Toxoplasma* IgG antibody determination performed [*n* (%)]	53 (98)
Positive [*n* (%)]	51 (96)
Serum concentration, mean [IQR] (IU/ml)	773 [197–1118]
Negative [*n* (%)]	1 (2)
Unknown [*n* (%)]	1 (2)
Lumbar punction performed [*n* (%)]	22 (35)
CSF PCR *T. gondii* performed [*n* (%)]	6 (27)
Positive [*n* (%)]	1 (17)
Negative [*n* (%)]	5 (83)
CSF *Toxoplasma* IgG antibody determination performed [*n* (%)]	17 (77)
Positive [*n* (%)]	1 (6)
Negative [*n* (%)]	6 (35)
Unknown [*n* (%)]	10 (59)
Goldmann–Wittmer coefficient and albumin index performed [*n* (%)]	7 (32)
Goldmann–Wittmer coefficient, median [IQR]	1.1 [0.6–1.9]
Albumin index, median [IQR]	12.8 [5.3–13.4]
Brain biopsy obtained [*n* (%)]	11 (18)
Brain biopsy preceded HIV diagnosis [*n* (%)]	7 (64)
Biopsy PCR *T. gondii* performed [*n* (%)]	7 (64)
Positive [*n* (%)]	7 (100)
Negative [*n* (%)]	0 (0)

CSF, cerebrospinal fluid; IQR, interquartile range.

Lumbar punction was performed in 35% (*n* = 22) of patients. PCR for *T. gondii* in CSF was performed in six (27%) of those patients, and all but one were negative. Serological examination to detect enrichment of *Toxoplasma*-specific IgG antibodies in CSF was performed in 77% (*n* = 17) of those patients. Data on performed serology was unknown in majority of patients (*n* = 10, 59%) due to poor registration. Data of remaining seven (41%) patients showed a single positive GWC and negative examination in six remaining patients. The mean GWC's and albumin index to examine the intactness of blood–brain barrier were, respectively 1.1 (0.6–1.9), meaning negative, and 12.8 (5.3–13.4), meaning slight permeability.

Brain biopsy was performed in 18% (*n* = 11) of patients. In seven patients, brain biopsy preceded HIV diagnosis, whilst in four, brain biopsy was performed whilst patients were known to be HIV-positive. In these four patients, biopsies were taken to exclude localization of cerebral lymphoma alongside cerebral toxoplasmosis, as three out of four patients were not responding to antitoxoplasma therapy and the remaining one patient needed brain surgery for decompression salvage therapy. PCR for *T. gondii* on acquired tissue was performed in seven patients, and all were positive.

### Treatment

Initial induction therapy consisted in most patients (*n* = 41, 65%) of pyrimethamine, sulfadiazine, and folinic acid (P-S) (Table 4). Cotrimoxazole (TMP-SMX) monotherapy or combined with pyrimethamine and folinic acid was used in 12 (19%) patients as initial therapy. Five (8%) patients were initially treated with pyrimethamine, clindamycin, and folinic acid (P-C). Initial induction therapy of four (6%) patients was unknown. In one patient, treatment was not started due severe brain compression, and palliative care was directly initiated.

**Table 4:** Treatment information and outcomes of people with HIV with cerebral toxoplasmosis.

Treatment (*n* = 63)	
Initial induction therapy [*n* (%)]	62 (98)
Sulfadiazine, pyrimethamine, folinic acid [*n* (%)]	41 (65)
Clindamycin, pyrimethamine, folinic acid [*n* (%)]	5 (8)
Cotrimoxazole monotherapy or combined pyrimethamine, folinic acid [*n* (%)]	12 (19)
Unknown [*n* (%)]	4 (6)
Course of treatment	
Continuation of initial induction therapy [*n* (%)]	38 (60)
Switch of initial induction therapy [*n* (%)]	19 (30)
Time to switch induction therapy, mean [IQR] (days)	15 [6–20]
Continuation of secondary treatment [*n* (%)]	15 (80)
Switch of secondary treatment [*n* (%)]	4 (21)
Unknown [*n* (%)]	3 (5)
Discontinuation of treatment [*n* (%)]	2 (3)
Duration of induction therapy, mean [IQR] (days)	51 [42–52]
Additional treatment with corticosteroids [*n* (%)]	14 (22)
Secondary prophylaxis started [*n* (%)]	38 (60)
Duration secondary prophylaxis, mean [IQR] (months)	8 [5–10]
Decompressive craniotomy [*n* (%)]	1 (2)

IQR, interquartile range.

Initial induction therapy was continued in 60% (*n* = 38) of patients, and therapy was switched in 30% (*n* = 19) of patients. In three (5%) patients, no data were available after initial induction therapy was started. In two (3%) patients, therapy was started because of a high suspicion of cerebral toxoplasmosis due to papillary oedema and positive *Toxoplasma* IgG antibody determination in blood and liquor, but they lacked abnormalities on imaging. Eventually, the diagnosis cerebral toxoplasmosis was rejected for those patients without conclusive alternative diagnosis, and their therapy was stopped early after 9 and 12 days. Switch of induction therapy was mostly due to side effects, except for one patient who was suspected of therapeutic failure as defined by clinical deterioration and progression of lesions on imaging. Most common reported side effects were fever (*n* = 6), rash (*n* = 5), cytopenia (*n* = 4), liver failure (*n* = 3), and nausea (*n *= 2). Fifteen patients completed their second-line treatment regimen, three patients switched yet another time due to side effects, and the same one patient for which the initial treatment failed switched again due to presumed therapeutic failure. Patients were on induction therapy for a mean of 51 days. Subsequently, secondary prophylaxis was given to 38 (60%) patients for a mean of 8 months.

A minority of patients (*n* = 14, 22%) had received additional treatment with corticosteroids during induction therapy. Decompressive craniotomy was performed in one patient.

### Clinical outcomes

**Fig. 2:**
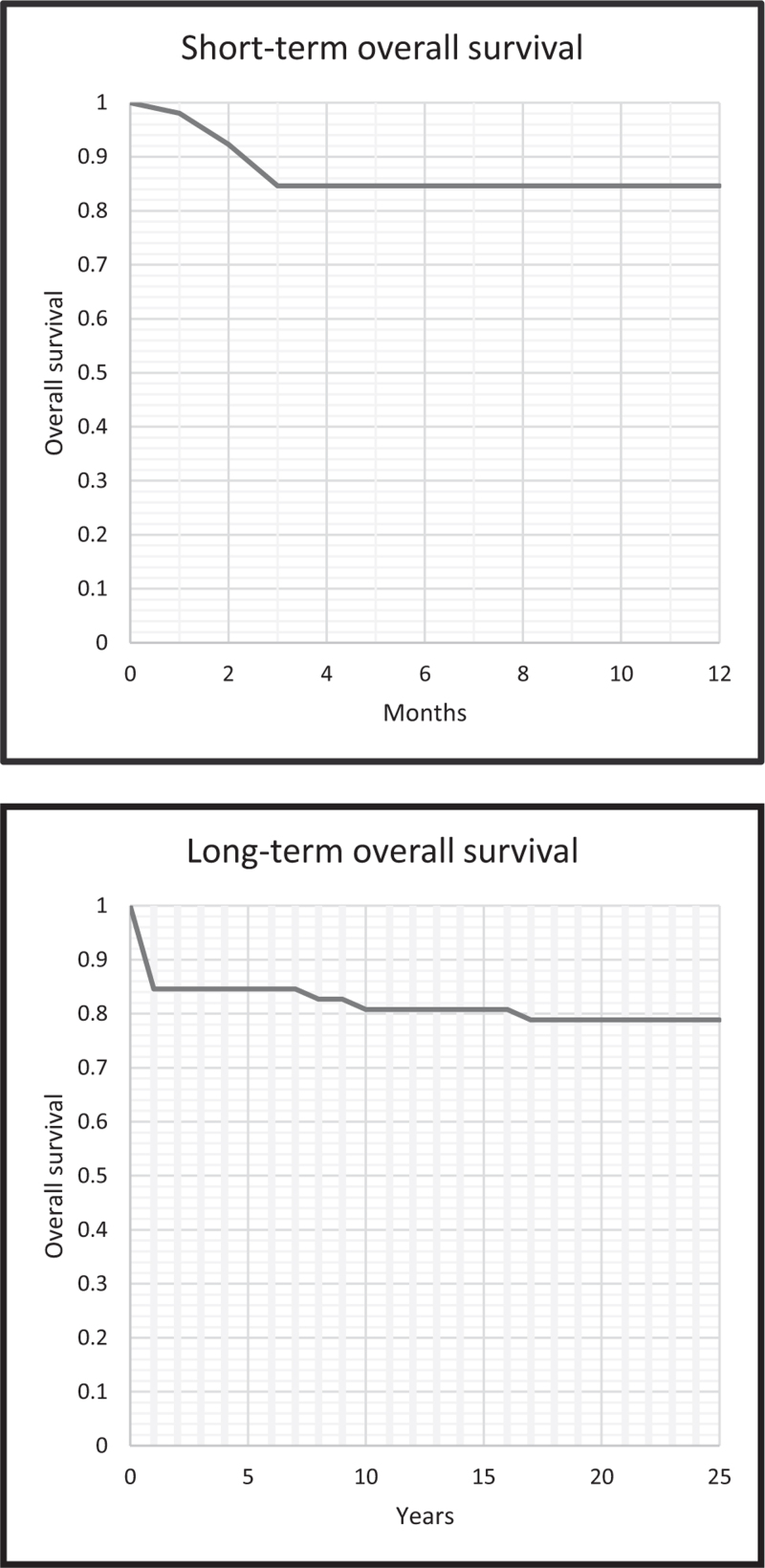
Kaplan–Meier survival curve for overall survival of people with HIV with cerebral toxoplasmosis (a) in the first year and (b) by years after cerebral toxoplasmosis diagnosis.

Mean patient follow-up after cerebral toxoplasmosis diagnosis was 15 years (Table 5). Short-term and long-term overall survival is illustrated using Kaplan–Meier curves (Fig. 2a and b). End-point was reached if a patient had passed away or was lost to follow-up. Missing data from patients (*n* = 11, 17%), who were early lost to follow-up were left out. Most patients (*n* = 41, 79%) were still alive at the end of inclusion. Of the 11 patients who died, 8 died within the first 3 months of diagnosis directly related to AIDS and their cerebral toxoplasmosis diagnosis. The other three patients died 8, 10, and 17 years after diagnosis due to HIV-unrelated disease.

**Table 5:** Clinical outcomes of people with HIV with cerebral toxoplasmosis within the first year of diagnosis.

Clinical outcomes (*n* = 63)	
ICU admission [*n* (%)]	7 (11)
Duration ICU stay, mean [IQR] (days)	21 [16–29]
Intubation [*n* (%)]	3 (5)
Circulatory support [*n* (%)]	2 (3)
Alive after time of diagnosis	
1 week [*n* (%)]	51 (81)
1 month [*n* (%)]	48 (76)
3 months [*n* (%)]	44 (70)
6 months [*n* (%)]	44 (70)
1 year [*n* (%)]	44 (70)
Unknown [*n* (%)]	11 (17)
Glasgow Coma Score after time of diagnosis	
1 week, mean [IQR]	15 [15–15]
1 month, mean [IQR]	15 [15–15]
3 months, mean [IQR]	15 [15–15]
6 months, mean [IQR]	15 [15–15]
1 year, mean [IQR]	15 [15–15]
Ability to walk after time of diagnosis	
1 week [*n* (%)]	57 (85)
1 month [*n* (%)]	60 (92)
3 months [*n* (%)]	57 (92)
6 months [*n* (%)]	52 (93)
1 year [*n* (%)]	51 (93)
State of living after 1 year of diagnosis	
Independent [*n* (%)]	40 (64)
Assisted living [*n* (%)]	2 (3)
Nursing home [*n* (%)]	2 (3)
Follow-up time after diagnosis, mean [IQR] (years)	15 [11–20]

IQR, interquartile range.

Most patients (*n* = 56, 89%) were cared for at a nursing ward, whereas seven patients needed intensive care admission for mechanical ventilation or circulatory support. The number of patients that were still in follow-up and able to walk independently at 1, 6, and 12 months after diagnosis was 60 (92%), 52 (93%) and 51 (93%), respectively. Of those alive 12 months after diagnosis, a minority of patients were residing in a nursing home or at home with supportive care (*n* = 4).

## Discussion

Our patient population included patients between 2000 and 2024, compromising almost 25 years of cerebral toxoplasmosis in PWH in a large university medical centre in the Netherlands. In our patient population, cerebral toxoplasmosis in PWH was predominantly diagnosed in middle-aged men, corresponding with the distribution of PWH in the Netherlands [17]. Seventy-one percent (*n* = 45) of patients with cerebral toxoplasmosis was not born in the Netherlands, which corresponds to the distribution of HIV within the general population of the Netherlands and the worldwide distribution of seroprevalence of toxoplasmosis [5–7,17].

Almost all patients with cerebral toxoplasmosis were identified as late presenters. Half of new HIV patients (52%) in the Netherlands are late presenters, despite all efforts for early diagnosis in the last decades [17]. In most of our patients, cerebral toxoplasmosis was their first presenting sign of HIV. In a fourth of patients, cerebral toxoplasmosis developed months to years following their HIV diagnosis, mainly due to cART not yet being initiated, halted, or initially refused. This partly reflects historic HIV treatment guidelines, in which initiation of cART was postponed until a decline in CD4^+^ T-cell count was noted, and it reflects difficulties in adherence in general. Of interest, is that a part of patients presented with cerebral toxoplasmosis as unmasking IRIS after recent start of cART. Their clinical presentation and diagnostic findings were comparable to other patients. However, their mean CD4^+^ T-cell count increased more rapidly in the first months after initiation of cART, potentially rendering them at higher risk for developing IRIS. IRIS is not common for cerebral toxoplasmosis, unlike *Mycobacterium tuberculosis* or *Cryptococcus neoformans*, and has only been described in a handful of case reports [18,19].

Clinicians should, therefore, consider cerebral toxoplasmosis in late presenters with neurological symptoms or lesions on imaging, and more specifically when already known PWH present with new neurological symptoms in the first months after initiating cART.

Cerebral toxoplasmosis in PWH can present with a broad spectrum of clinical manifestations. Although most patients presented with headache, neurological symptoms, and fever, a small group had only aspecific symptoms such as fatigue and nausea, warranting a high index of suspicion of cerebral toxoplasmosis in all PWH with a low CD4^+^ T-cell and aspecific symptoms.

In our cohort, IgG for toxoplasmosis in serum was positive in all but one patient, whereas *Toxoplasma* IgM was absent in all patients, suggesting reactivation of an earlier acquired infection.

Although we observed high IgG titres in most patients in our cohort, they have low predictive value as microbiologic standalone test, especially in patients with severe immune deficits, such as AIDS [20,21]. Comparing ratios of serological results in blood and liquor, thereby demonstrating specific antibody production in liquor, using a GWC or Reiber index, remains an important addition in diagnosing CNS toxoplasmosis. Additional testing for the presence of *T. gondii* DNA in CSF should also be performed, albeit with a variably known low sensitivity for PCR in CSF in PWH (50–98%) [22–24].

The low number of patients with CNS toxoplasmosis for which a positive PCR was found in this study has several explanations. First, PCR on *T. gondii* was implemented in our hospital in 2005, and over half of the CNS toxoplasmosis patients in our cohort was diagnosed before implementation of the PCR on *T. gondii* (between the year 2000 and 2006). Of the 33 included patients after implementation of the PCR on *T. gondii*, a lumbar puncture could only be performed in 10 of these patients, and our PCR method was used in six samples, in which *T. gondii* DNA was detected in only one of those six samples (17%). This low sensitivity of the PCR examination used in our hospital is not caused by a poor efficiency of our PCR method, as our method performed well in the external quality assessment schemes from the Quality Control for Molecular Diagnostics (QCMD). Therefore, it is likely that the amount of *T. gondii* DNA in CSF obtained by lumbar punctures is rather low in PWH with CNS toxoplasmosis. This could be explained by the relatively low number of *T. gondii* tachyzoites released from lysed cells compared to viral and bacterial microbes that can cause meningitis. PCR on brain tissue was positive in 100% of tests performed.

Although PCR for the presence of *T. gondii* DNA in blood may seem an attractive alternative, patients with cerebral toxoplasmosis may have a low concentration of *T. gondii* DNA in blood, which appears to negatively affect the sensitivity [1,25,26]. The severity of the cerebral toxoplasmosis seems to be related to the performance of the *T. gondii* PCR on blood [25,26].

Brain biopsy was performed in 11 patients. In seven of those patients, biopsies were performed prior to the HIV diagnosis. In the remaining four patients, biopsies were taken to exclude localization of cerebral lymphoma due to lack of response after initiation of antitoxoplasma therapy or need for surgical decompression for elevated intracranial pressure.

So, whilst pleading for a low threshold of HIV testing in patients with brain lesions can potentially prevent brain biopsies and associated morbidity and mortality of the procedure in the future, brain biopsy should still be considered in patients who are already undergoing surgical decompression for elevated intracranial pressure, in patients who do not show clinical improvement within 14 days of anti-*Toxoplasma* therapy, or in patients in whom diagnosis remains inconclusive.

In literature, MRI is deemed more sensitive than CT for radiological diagnosis of cerebral toxoplasmosis and similar in specificity [1,27,28]. However, in our cohort in patients who underwent both a CT scan with contrast and an MRI scan, images were comparable. CT is a more common available imaging technique than MRI in most hospitals worldwide [29–31].

Therefore, clinicians can consider both MRI scan or CT scan with contrast for diagnosing cerebral toxoplasmosis.

Initial induction therapy consisted in most patients of P-S, which is the first-choice therapy in the Netherlands and worldwide [1,12,32–35]. Some patients were treated with TMP-SMX monotherapy or combined pyrimethamine and foline acid due to the intravenous option of administration or limited availability of first-choice therapy. Available data suggest a similar rate of clinical response and risk of adverse events between P-S and TMP-SMX [36]. Two-thirds of patients were able to continue and finish initial induction therapy. On the other hand, one-third of patients had to switch initial induction therapy, mostly due to commonly known side effects such as fever and rash. However, most of these patients completed secondly initiated induction therapy.

Patient overall survival in this cohort is promising after the initial phase of induction therapy. However, almost 10% of patients were living in a nursing home or at home with supportive care at 12-month mark, illustrating that the rate of neurological long-term complications due to cerebral toxoplasmosis continues to be high and to have a significant impact on quality of life.

In conclusion, cerebral toxoplasmosis remains the most common cause of central nervous system infection in PWH and continues to cause high morbidity and mortality. Cerebral toxoplasmosis presents itself with a broad spectrum of clinical manifestations and variable patterns on imaging. High index of suspicion by clinicians is vital to timely start induction therapy and therewith attempt to limit long-term neurological complications and mortality. Identifying PWH earlier could reduce incidence of cerebral toxoplasmosis due to timely start of cART and anti-*Toxoplasma* prophylaxis.

## Acknowledgements

C.M.v.D. collected all data. C.M.v.D. and E.v.N. contributed equally to reviewing all data and writing the manuscript. E.v.N. was principal investigator. J.J.v.H. contributed to writing the manuscript. B.J.A.R., H.I.B., C.R., T.E.M.S.d.V., C.A.M.S., J.L.N., M.d.M.-M., A.A.A., L.S., and E.C.M.v.G. reviewed the manuscript.

### Conflicts of interest

There are no conflicts of interest.
